# Study on the Predictive Value of a Pulmonary Edema Imaging Score for Delayed Extubation in Patients after Heart Valve Surgery on Cardiopulmonary Bypass

**DOI:** 10.31083/j.rcm2510387

**Published:** 2024-10-28

**Authors:** Xuefeng Lin, Funan Wang, Yuting Wang

**Affiliations:** ^1^Department of Critical Care Medicine, Zhongshan Hospital (Xiamen), Fudan University, 361015 Xiamen, Fujian, China; ^2^Department of Radiology, Zhongshan Hospital (Xiamen), Fudan University, 361015 Xiamen, Fujian, China

**Keywords:** cardiac valve surgery, delayed extubation, RALE score, predictive value

## Abstract

**Background:**

Delayed extubation with mechanical ventilation after cardiac valve surgery is an important clinical challenge. Early extubation can improve the survival rate and prognosis of patients. The study aims to explore the predictive value of a chest X-ray pulmonary edema imaging score on the first day after surgery for delayed extubation in patients after cardiac valve surgery on cardiopulmonary bypass.

**Method:**

Retrospective analysis of the clinical data of patients undergoing cardiac valve surgery under extracorporeal circulation admitted to the intensive care unit of Zhongshan Hospital Affiliated with Fudan University (Xiamen) from January 2020 to October 2023. The patients were divided into an early extubation group according to the postoperative mechanical ventilation time (time: <24 h) and a delayed extubation group (time: ≥24 h). The radiographic assessment of lung edema (RALE) score was performed on the chest X-ray of the patient on the first day after surgery to analyze the correlation between delayed extubation of mechanical ventilation and the chest radiograph RALE score on the first day after surgery and to verify its predictive performance.

**Results:**

Significant differences in age, the incidence of hypertension, body mass index (BMI), left ventricular ejection fraction (LVEF), pump time, RALE score, ventilation time, oxygenation index, P_a_CO_2_, and brain natriuretic peptide (BNP) levels after the first 24 h were seen between patients who were extubated before and 24 h post operation (*p* = 0.013, 0.001, 0.034, <0.001, <0.001, <0.001, <0.001, <0.001, 0.014, and <0.001, respectively). No significant differences were observed in the proportion of males and the lactate level after the first 24 h between the two groups (*p* = 0.792 and 0.191, respectively). The time of mechanical ventilation was positively correlated with the RALE score in all patients, and the correlation coefficient was 0.419; the difference was statistically significant (*p* < 0.001). Multivariate binary logistic regression analysis with stepwise regression was performed on each research factor, and it was found that RALE score, pump time, oxygenation index, and postoperative BNP were independent risk factors for predicting delayed extubation in patients undergoing cardiac surgery on cardiopulmonary bypass. A 10-fold cross-validation revealed that the mean accuracy, sensitivity, specificity, and area under the curve (AUC) of the regression model were 0.737, 0.749, 0.741, and 0.825, respectively.

**Conclusions:**

The RALE score on the chest radiograph on the first day after surgery is an independent risk factor for predicting delayed extubation in patients after cardiac valve surgery on cardiopulmonary bypass and has good predictive value.

## 1. Introduction

Although significant progress has been made in cardiac surgery technology and anesthesia management, delayed extubation during postoperative mechanical ventilation remains a common problem after heart valve surgery. Studies [1, 2] have shown that a significant proportion of patients (2.6%–30%) experience prolonged ventilation after cardiac surgery. Patients with prolonged mechanical ventilation have a high in-hospital mortality and are associated with early and mid-term complications and a significant reduction in survival [2, 3]. In a study involving nearly 2000 post-cardiac surgery patients, Hessels *et al*. [4] found that 11% required mechanical ventilation for more than 24 hours. Furthermore, they noted that mortality rates increased as the duration of mechanical ventilation was prolonged [4]. Therefore, it is important to develop reliable indicators for predicting delayed extubation after cardiac surgery to help the formulation of surgical plans and the rational utilization and management of postoperative resources to reduce mortality in these patients [5, 6].

In 2018, Warren *et al*. [7] proposed the radiographic assessment of lung edema (RALE) score to quantify the severity of lung lesions on chest X-rays and confirmed that, in acute respiratory distress syndrome (ARDS), the RALE score could be used to assess the severity of pulmonary edema and ARDS and that this was related to the patient’s clinical outcome. It has also been demonstrated that the RALE score is related to early weaning and extubation in patients with ARDS and can predict the duration of mechanical ventilation [8]. Therefore, the RALE score is a highly reproducible tool that can be easily implemented at the bedside and quantifies the extent of radiographic pulmonary edema with high reliability. This study retrospectively analyzed the correlation between delayed extubation in patients after cardiac valve surgery and the chest radiograph RALE score on the first day after surgery and explored whether the RALE score can be used to predict delayed extubation and provide clinical guidance for extubation in patients undergoing cardiac valve surgery.

## 2. Methods

### 2.1 Research Patients

The clinical data of patients admitted to the intensive care unit of Zhongshan Hospital Affiliated with Fudan University (Xiamen) from January 2020 to October 2023 after cardiac valve surgery under extracorporeal circulation were collected. According to the postoperative mechanical ventilation time, the patients were divided into an early extubation group (time: <24 h) and a delayed extubation group (time: ≥24 h). Inclusion criteria: age >18 years; patients undergoing heart valve surgery under extracorporeal circulation. Exclusion criteria: patients who did not receive tracheal intubation and received mechanical ventilation when admitted to intensive care unit (ICU); patients who did not receive extracorporeal circulation during surgery; patients who did not have a chest X-ray on the first day after surgery; patients who underwent tracheotomy. This study was reviewed by the Ethics Committee of Xiamen Hospital, Zhongshan Hospital, Fudan University, review number B2021-037R(1). All participants provided written informed consent before enrollment.

### 2.2 Data Collection

Age, gender, weight, height, brain natriuretic peptide (BNP), oxygen partial pressure (PO_2_), inspired oxygen concentration (FiO_2_), oxygenation index (PO_2_/FiO_2_ ratio), carbon dioxide partial pressure (P_a_CO_2_), lactic acid (mmol/L), pump time (min), preoperative left ventricular ejection fraction (%), postoperative intubation mechanical ventilation time (h), chest X-ray image on the first day after surgery. Data were collected from electronic patient charts. The baseline information and relevant data of the patients are shown in Table 1.

**Table 1.** **Clinical data of the cohort**.

Variates	Overall	Delayed extubation	Early extubation	*p*-value
n	237	105	132	
Age (y)	57.00 (48.00, 67.00)	60.00 (50.00, 68.00)	55.00 (47.00, 64.25)	0.013
Male (n (%))	123 (51.9)	56 (53.3)	67 (50.8)	0.792
Hypertension (n (%))	66 (27.8)	41 (39.0)	25 (18.9)	0.001
BMI (kg/m^2)^	22.86 (20.50, 25.61)	23.53 (21.48, 26.04)	22.68 (20.20, 24.76)	0.034
LVEF (%)	63.00 (57.00, 67.00)	61.00 (54.00, 64.00)	65.00 (60.00, 69.00)	<0.001
Pump time (min)	143.00 (114.00, 178.00)	160.00 (127.00, 200.00)	130.50 (106.75, 159.25)	<0.001
RALE score	11.00 (9.00, 15.00)	13.00 (11.00, 18.00)	10.00 (7.00, 13.25)	<0.001
Ventilation time (h)	21.00 (17.00, 44.00)	49.00 (37.00, 110.00)	18.00 (16.00, 20.00)	<0.001
Postoperative BNP (pg/mL)	843.00 (415.00, 1554.00)	1164.00 (647.00, 1930.00)	636.00 (337.17, 1126.25)	<0.001
Oxygenation index	284.00 (206.00, 362.00)	254.00 (172.00, 350.00)	312.00 (234.00, 388.50)	<0.001
P_a_CO_2_ (mmHg)	36.00 (33.00, 40.00)	38.00 (33.00, 41.00)	35.00 (32.00, 39.00)	0.014
Lactic acid (mmol/L)	3.00 (2.10, 4.30)	3.30 (2.00, 4.80)	2.90 (2.10, 4.00)	0.191

BMI, body mass index; LVEF, left ventricular ejection fraction; RALE, radiographic assessment of lung edema; BNP, brain natriuretic peptide.

### 2.3 RALE Score

**Fig. 1.**
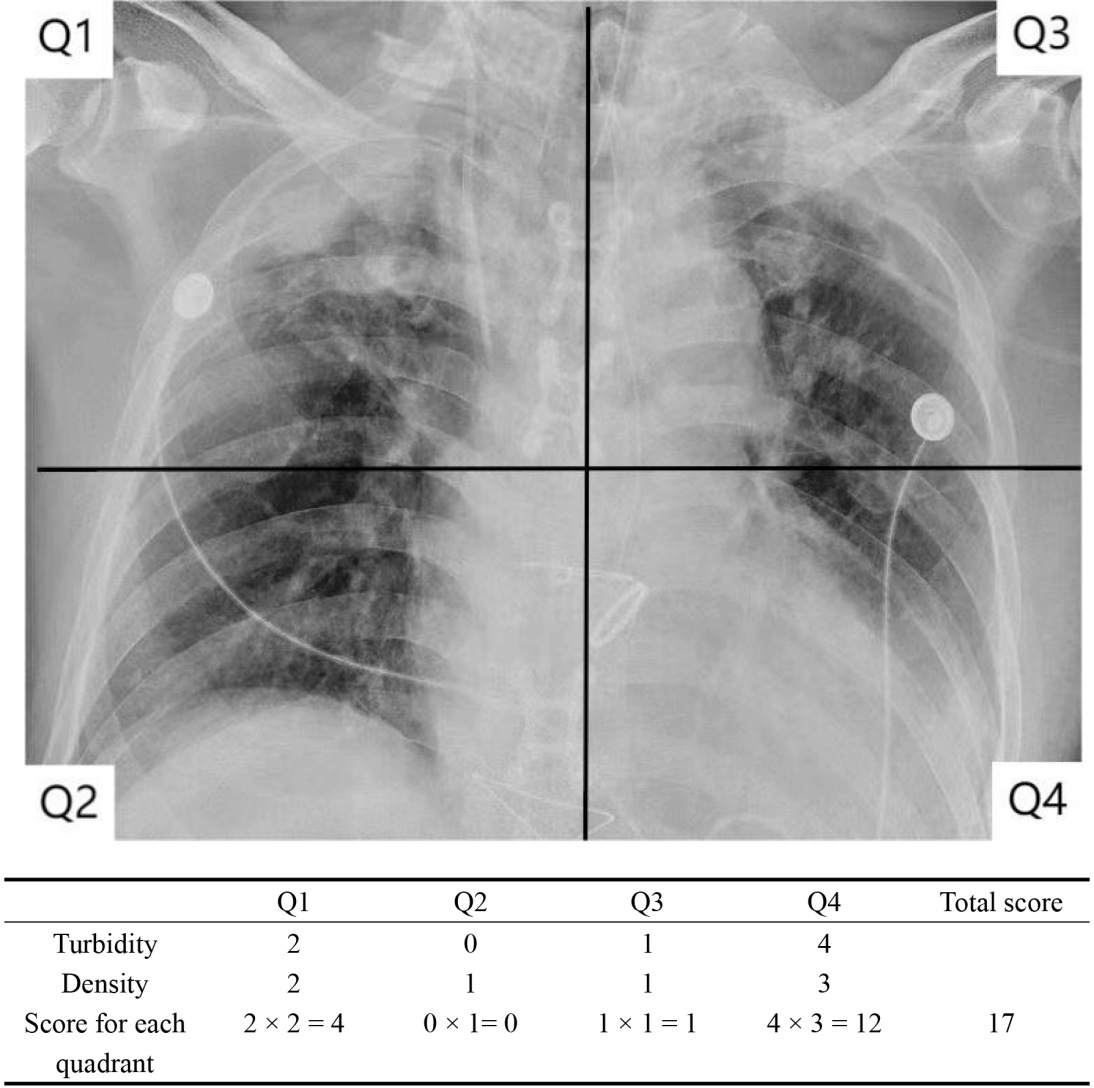
**Calculation of the RALE score**. RALE, radiographic assessment of lung edema.

The RALE score evaluates the degree and density of alveolar opacity on chest radiographs. The higher the score, the more serious the lung disease. Two senior imaging physicians independently scored each chest X-ray, and the average was taken as the RALE score result [7]. The chest X-ray is divided into four quadrants (Q1, Q2, Q3, Q4) according to the midline of the spine (longitudinal line) and the first bifurcation point of the left bronchus (horizontal line), see Table 2 and Fig. 1.

**Table 2.** **Calculation method of pulmonary edema imaging (RALE) score**.

Turbidity	
Turbidity score (Con)	Alveolar opacity
0	None
1	<25%
2	25%–50%
3	51%–75%
4	≥75%
Density	
Density score (Den)	Alveolar opacity density
1	Hazy
2	Medium
3	Dense
RALE rating	
Right lung	Left lung
Upper quadrant Q1 = Con × Den	Upper quadrant Q3 = Con × Den
Lower quadrant Q2 = Con × Den	Lower quadrant Q4 = Con × Den
RALE score = Q1 + Q2 + Q3 + Q4	

RALE, radiographic assessment of lung edema.

### 2.4 Statistical Analysis

All statistical analyses were performed using R Statistical Software (version 4.2.3, R Foundation for Statistical Computing, Vienna, Austria). Data are expressed as the median (interquartile range (IQR)) or number and percentage. All data were initially analyzed using the Kolmogorov–Smirnov test to assess for normality. When appropriate, quantitative variables were compared using the Chi-squared test or Fisher’s exact test. Qualitative variables were compared using Student’s *t*-test and Mann–Whitney U test for numerical variables. Multivariate binary logistic regression analysis with stepwise regression was used to construct a prediction model of risks for delayed extubating in patients after cardiopulmonary bypass surgery; then, a nomogram was drawn. A 10-fold cross-validation was used to assess the model’s generalization ability, which involves randomly dividing the original dataset into approximately equal-sized subsets of samples. Each subgroup was alternately combined into a training set comprising 9 subsets, while the remaining subset served as the test set. Evaluation metrics such as accuracy, sensitivity, specificity, and area under curve (AUC) are then calculated. All *p*-values were two-tailed, and *p* < 0.05 was considered statistically significant.

## 3. Results

**Fig. 2.**
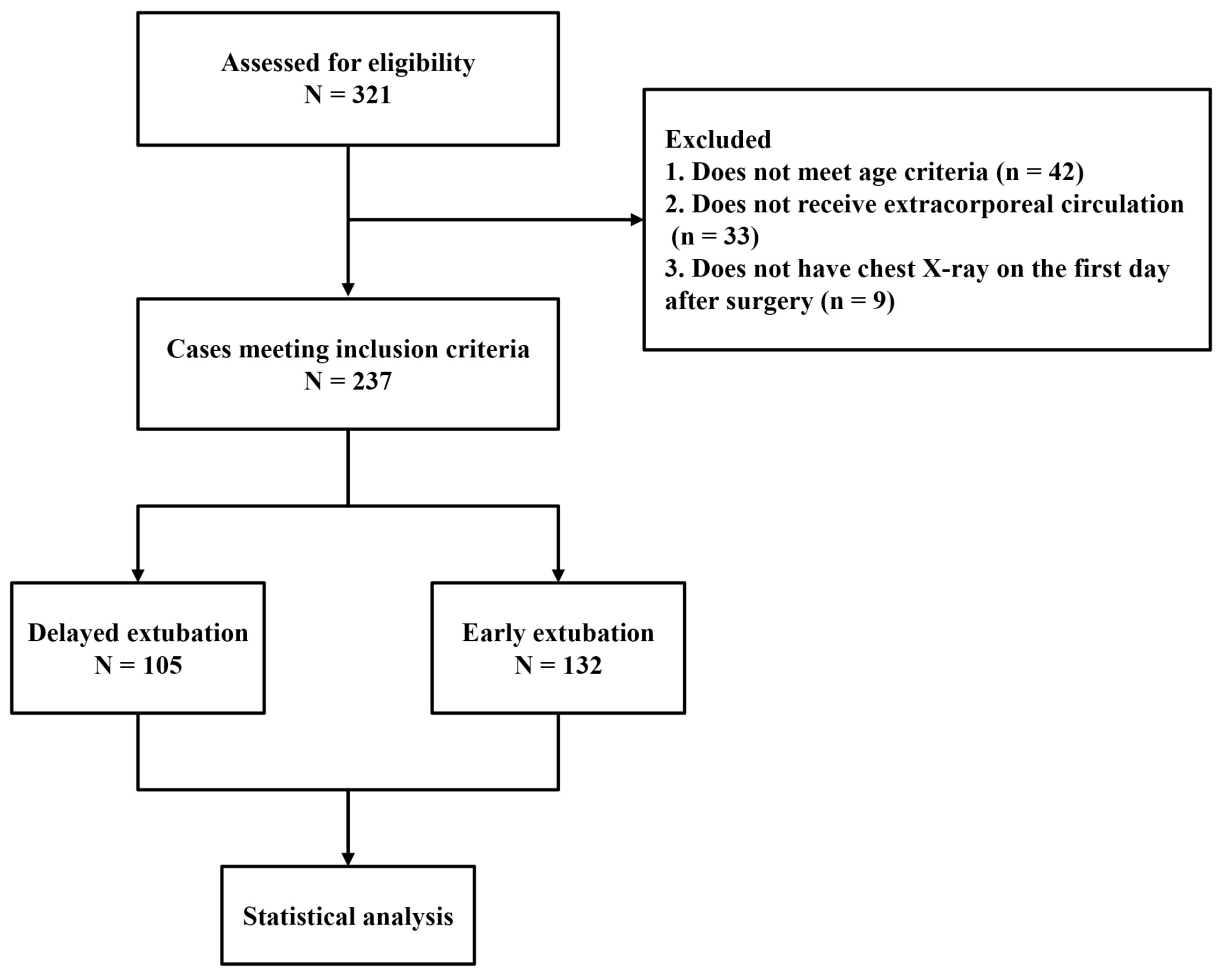
**Flow chart of the study**.

A total of 237 patients were enrolled in the study, including 123 males and 114 females (see Fig. 2). Significant differences in age, prevalence of hypertension, body mass index (BMI), left ventricular ejection fraction (LVEF), pump time, RALE score, ventilation time, oxygenation index, P_a_CO_2_ and BNP level after the first 24 h were seen between patients who were extubated before and 24 h following surgery (*p* = 0.013, 0.001, 0.034, <0.001, <0.001, <0.001, <0.001, <0.001, 0.014 and <0.001, respectively). No significant differences were observed in the proportion of males and the lactate level after the first 24 h between the two groups (*p* = 0.792 and 0.191, respectively). A comprehensive list of variables is presented in Table 1.

**Fig. 3.**
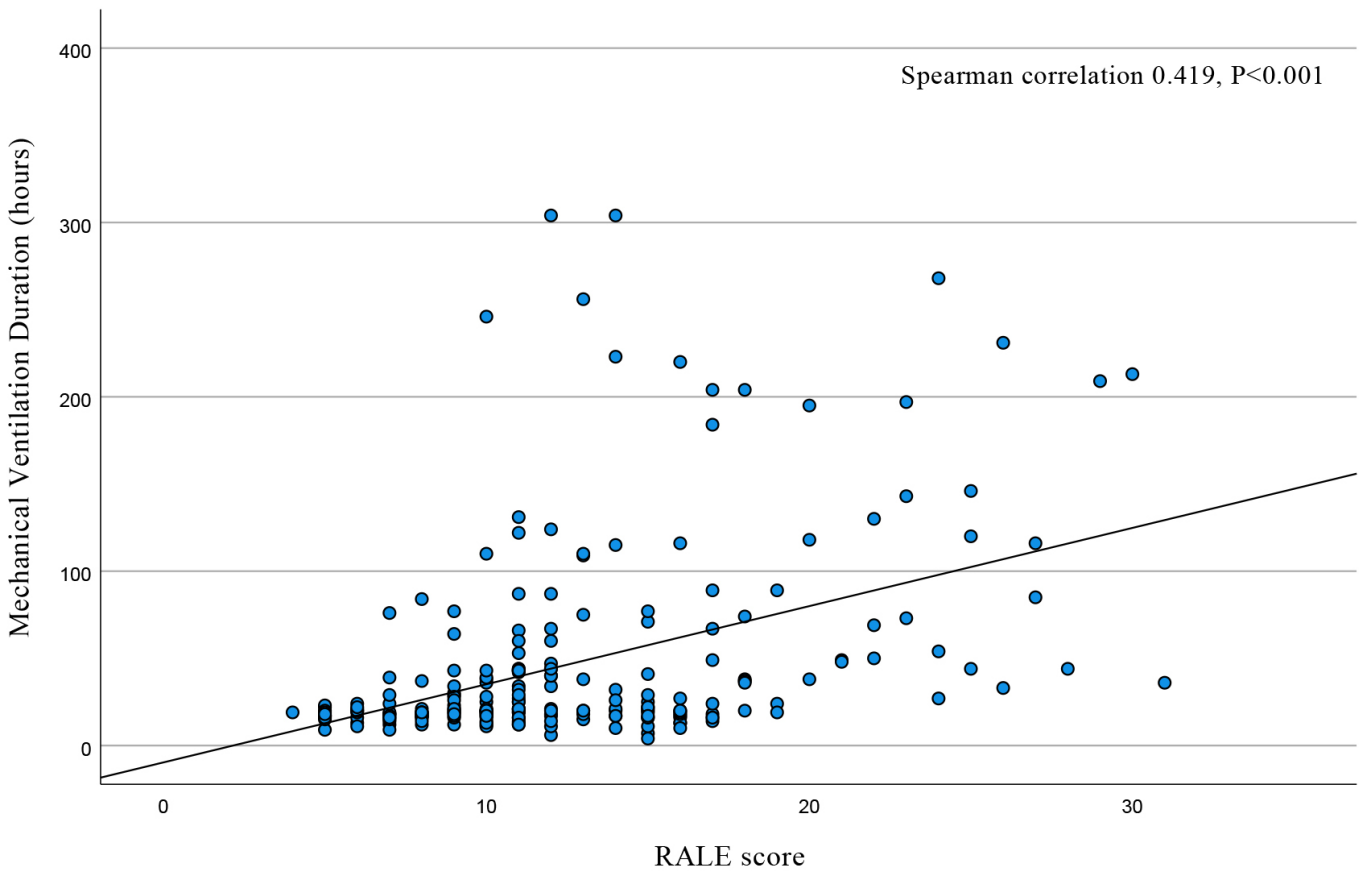
**Scatter plot of mechanical ventilation time and RALE score**. RALE, radiographic assessment of lung edema.

Spearman’s correlation analysis was performed on mechanical ventilation time and RALE score of all patients. The results showed that mechanical ventilation time was positively correlated with the RALE score, and the correlation coefficient was 0.419, with statistical significance (*p* < 0.001), as illustrated in Fig. 3.

**Fig. 4.**
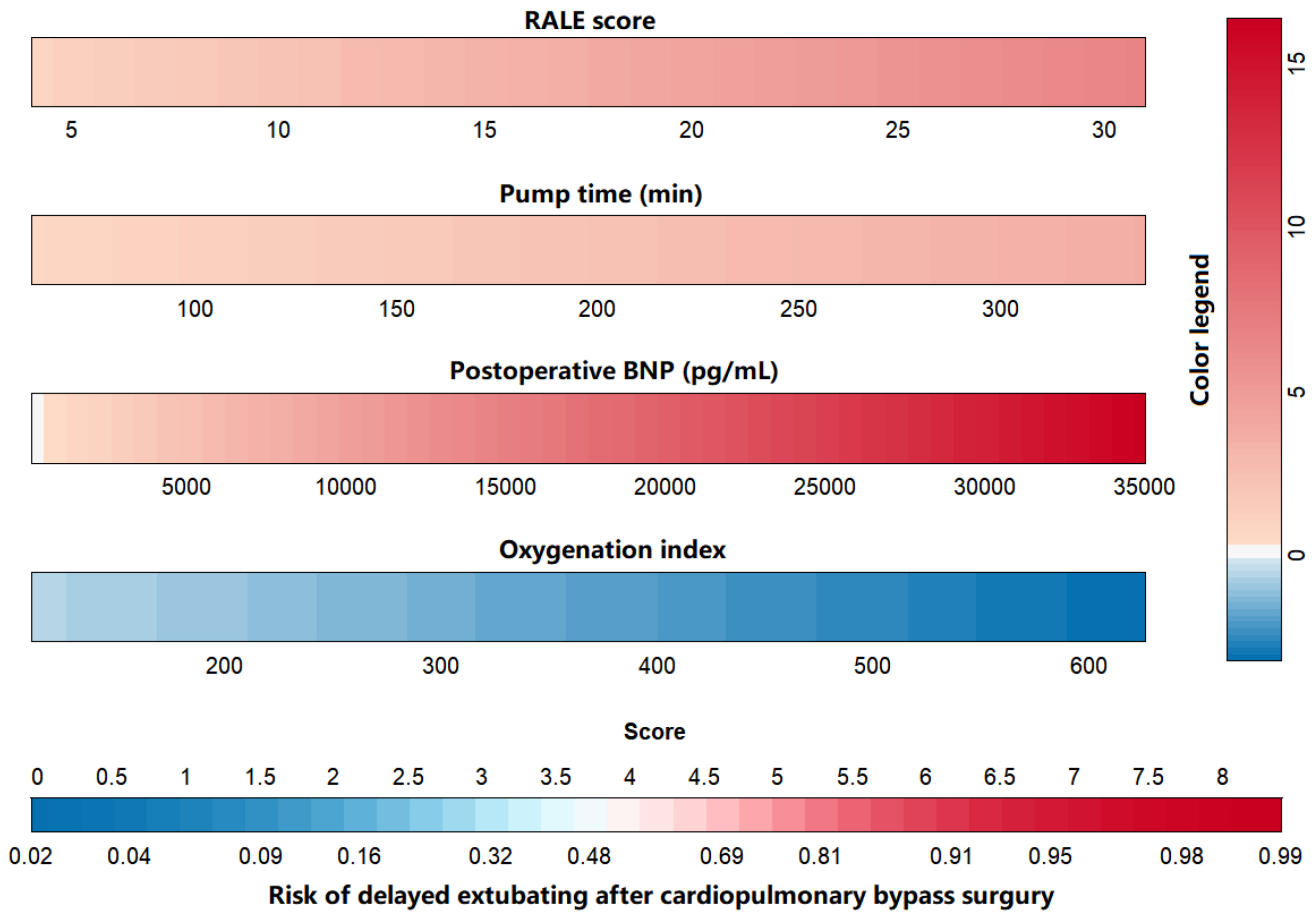
**Nomogram of delayed extubation risks in patients after cardiopulmonary bypass-assisted cardiac surgery**. RALE, radiographic assessment of lung edema; BNP, brain natriuretic peptide.

Multivariate binary logistic regression analysis with stepwise regression was performed on each research factor, and it was found that RALE score, pump time, oxygenation index, and postoperative BNP were independent risk factors for predicting delayed extubation in patients after cardiopulmonary bypass assisted cardiac surgery, listed in Table 3. The nomogram of the regression model is shown in Fig. 4.

**Table 3.** **Logistic analysis of risks for delayed extubating in patients after cardiac bypass-assisted cardiac surgery**.

Variates	OR	95% CI	Wald	*p*-value
RALE score	1.245	1.150–1.346	29.731	<0.001
Pump time	1.011	1.004–1.018	10.165	0.001
Oxygenation index	0.995	0.992–0.998	8.149	0.004
Postoperative BNP	1.000	1.000–1.001	10.241	0.001

OR, odds ratio; CI, confidence interval; RALE, radiographic assessment of lung edema; BNP, brain natriuretic peptide.

**Fig. 5.**
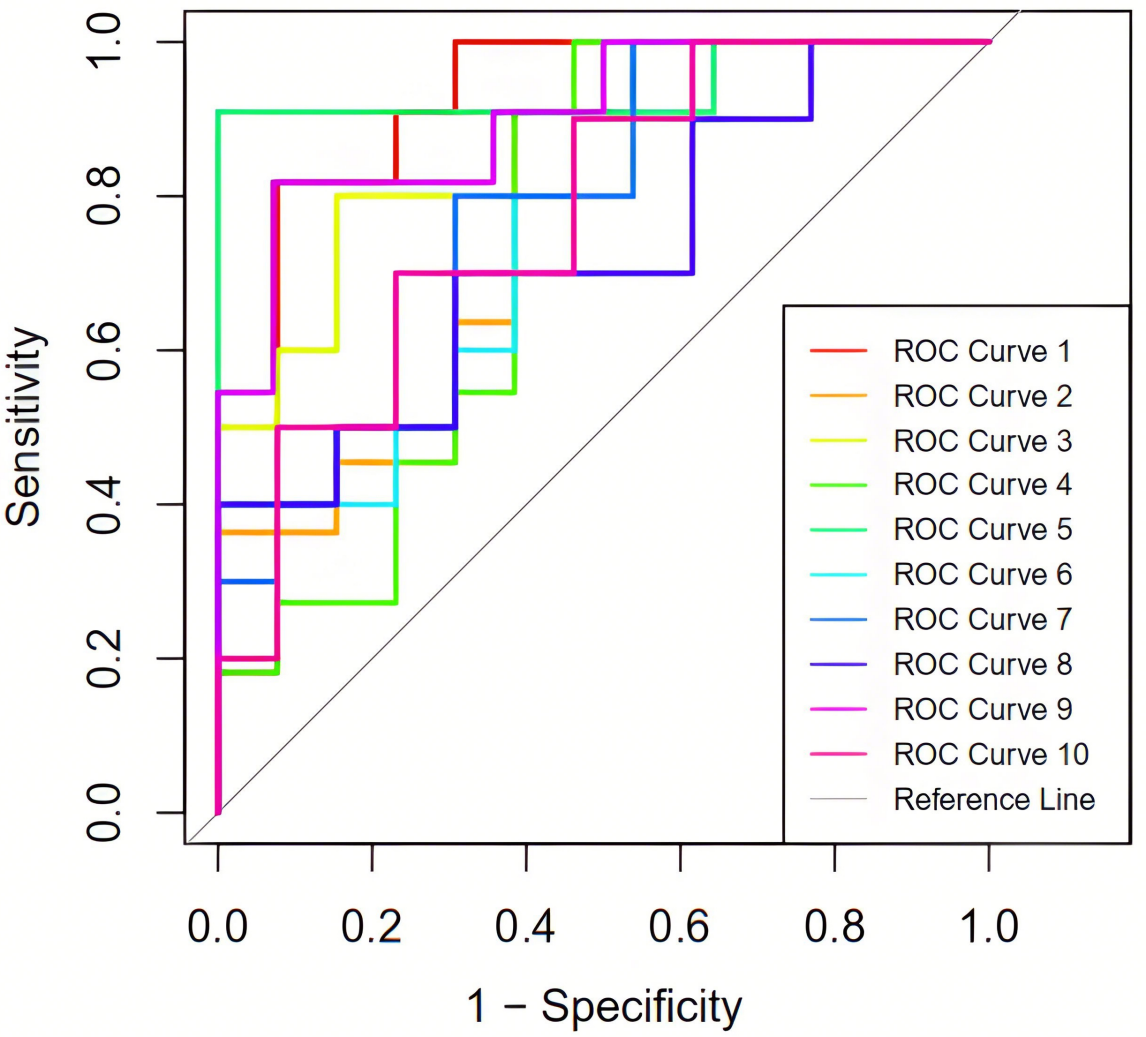
**The ROC curves under 10-fold cross-validation**. ROC, receiver operating characteristic.

A 10-fold cross-validation revealed that the mean accuracy, sensitivity, specificity, and AUC of the regression model were 0.737, 0.749, 0.741, and 0.825, respectively, as illustrated in Table 4 and Fig. 5.

**Table 4.** **The 10-fold cross-validation results and their mean value were obtained from 10 training iterations**.

Training set	Accuracy​	Sensitivity​	Specificity​	AUC
Fold-1 (ROC curve 1)	0.792	0.875	0.750	0.895
Fold-2 (ROC curve 2)	0.625	0.667	0.611	0.804
Fold-3 (ROC curve 3)	0.783	0.778	0.786	0.862
Fold-4 (ROC curve 4)	0.667	0.636	0.692	0.811
Fold-5 (ROC curve 5)	0.920	1.000	0.875	0.929
Fold-6 (ROC curve 6)	0.696	0.714	0.688	0.815
Fold-7 (ROC curve 7)	0.783	0.778	0.786	0.869
Fold-8 (ROC curve 8)	0.652	0.571	0.778	0.692
Fold-9 (ROC curve 9)	0.800	0.800	0.800	0.831
Fold-10 (ROC curve 10)	0.652	0.667	0.647	0.738
Average	0.737	0.749	0.741	0.825

AUC, area under the curve; ROC, receiver operating characteristic.

## 4. Discussion

Early extubation in patients after cardiac valve surgery can reduce perioperative complications, shorten intensive care unit duration and the total postoperative hospitalization time, improve the early and mid-term survival of patients, improve the patient’s prognosis, and reduce the medical burden [9, 10]. Recently, many studies have been conducted on the predictive factors of prolonged mechanical ventilation after cardiac surgery [11, 12, 13]. However, the correlation between the chest radiograph RALE score on the first day after cardiac valve surgery and delayed extubation has yet to be reported. Previously, the chest radiograph RALE score was mainly used to evaluate the severity and prognosis of pulmonary edema in patients with ARDS. Studies have confirmed that a continuously elevated RALE score positively correlates with prolonged mechanical ventilation time [7, 8].

Older age results in reduced physiological reserves and increased occurrence of underlying diseases. Previous studies have found that advanced age, gender, BMI >35 kg/m^2^, and left ventricular ejection fraction <30% are predictive factors for prolonged mechanical ventilation [14, 15]. Our results also show that age, obesity, and preoperative left ventricular ejection fraction are associated with delayed extubation.

Totonchi *et al*. [5] reported that increasing cardiopulmonary bypass time increases the risk of delayed extubation. Our study also showed that cardiopulmonary bypass time is an independent factor in delayed extubation after cardiac valve surgery. Prolonged extracorporeal circulation time increases the contact time between blood and exogenous substances, which leads to the activation of inflammatory mediators. This activation leads to lung damage and respiratory failure, thus prolonging the mechanical ventilation time [16].

The RALE score provides an innovative method to quantify the severity of lung injury by scoring the four quadrants of the lung based on opacity and density using routinely collected information from chest radiographs [17]. It allows for noninvasive assessment of the severity of pulmonary edema and lung infiltration, reflects the cardiopulmonary function of patients, and can predict whether patients can be extubated earlier. Jabaudon *et al*. [18] found that the RALE score was related to the severity of lung injury and survival in patients with ARDS. Kotok *et al*. [8] also demonstrated that the RALE score is associated with early weaning and extubation in patients with ARDS. Research has indicated that reducing radiological pulmonary edema consistently corresponds to clinical physiological improvement, facilitating early patient extubation. However, studies have not identified a significant correlation between baseline RALE scores and inflammation or metabolism. Our study found that the duration of mechanical ventilation in patients after heart valve surgery on cardiopulmonary bypass was positively correlated with the chest radiograph RALE score on the first day after surgery. Through cross-validation using the regression model, we confirmed that the RALE score is an independent risk factor for delayed extubation in patients with heart valve surgery on cardiopulmonary bypass and confirmed that the RALE score had high predictive efficacy in delayed extubation of patients after cardiopulmonary bypass (AUC value: 0.825).

### Study Limitations

Firstly, this study is a single-center, retrospective study with a small sample size, which may have a certain degree of bias. Therefore, future studies need to increase the sample size. Secondly, this study only analyzed potential risk factors of clinical concern and conducted binary and logistic regression analyses. However, it cannot be ruled out that other hidden risk factors may affect the results. Furthermore, the subjects included in this study were all patients who underwent valve replacement or repair with extracorporeal circulation. Thirdly, no comparator studies (lung sonography index and extravascular lung water index (ELWI)) were evaluated.

## 5. Conclusions

The RALE score on the chest radiograph on the first day after surgery is an independent risk factor for predicting delayed extubation in patients after cardiac valve surgery on cardiopulmonary bypass and has good predictive value.

## Availability of Data and Materials

The datasets for this study are available from the corresponding author upon reasonable request.
